# Spatial Heterogeneity and Peptide Availability Determine CTL Killing Efficiency *In Vivo*

**DOI:** 10.1371/journal.pcbi.1003805

**Published:** 2014-09-18

**Authors:** Thea Hogan, Ulrich Kadolsky, Sim Tung, Benedict Seddon, Andrew Yates

**Affiliations:** 1 Immune Cell Biology, MRC National Institute for Medical Research, Mill Hill, London, United Kingom; 2 Department of Systems and Computational Biology, Albert Einstein College of Medicine, New York, New York, United States of America; 3 Institute of Immunity and Transplantation, Division of Infection and Immunity, UCL, Royal Free Hospital, London, United Kingdom; 4 Department of Microbiology and Immunology, Albert Einstein College of Medicine, New York, New York, United States of America; 5 Institute of Infection, Immunity & Inflammation, College of Medical, Veterinary & Life Sciences, University of Glasgow, Glasgow, United Kingdom; La Jolla Institute for Allergy and Immunology, United States of America

## Abstract

The rate at which a cytotoxic T lymphocyte (CTL) can survey for infected cells is a key ingredient of models of vertebrate immune responses to intracellular pathogens. Estimates have been obtained using *in vivo* cytotoxicity assays in which peptide-pulsed splenocytes are killed by CTL in the spleens of immunised mice. However the spleen is a heterogeneous environment and splenocytes comprise multiple cell types. Are some cell types intrinsically more susceptible to lysis than others? Quantitatively, what impacts are made by the spatial distribution of targets and effectors, and the level of peptide-MHC on the target cell surface? To address these questions we revisited the splenocyte killing assay, using CTL specific for an epitope of influenza virus. We found that at the cell population level T cell targets were killed more rapidly than B cells. Using modeling, quantitative imaging and *in vitro* killing assays we conclude that this difference *in vivo* likely reflects different migratory patterns of targets within the spleen and a heterogeneous distribution of CTL, with no detectable difference in the intrinsic susceptibilities of the two populations to lysis. Modeling of the stages involved in the detection and killing of peptide-pulsed targets *in vitro* revealed that peptide dose influenced the ability of CTL to form conjugates with targets but had no detectable effect on the probability that conjugation resulted in lysis, and that T cell targets took longer to lyse than B cells. We also infer that incomplete killing *in vivo* of cells pulsed with low doses of peptide may be due to a combination of heterogeneity in peptide uptake and the dissociation, but not internalisation, of peptide-MHC complexes. Our analyses demonstrate how population-averaged parameters in models of immune responses can be dissected to account for both spatial and cellular heterogeneity.

## Author Summary

Measurements of the rates at which a single cytotoxic T lymphocyte (CTL) can survey for infected cells, and kill them upon encounter, are important for constructing predictive models of vertebrate immune responses to intracellular pathogens. The surveillance rate has been estimated previously using combinations of modeling and experiment, making the assumption that CTL and target cells are well-mixed and that all cell types are killed with equal efficiency. In this study we take an iterative approach with theory and experiment to go beyond such models and detail the effects of cellular heterogeneity, the spatial organisation of the tissue within which killing is taking place, and the influence of the level of expression of peptides on the target cell surface. We demonstrate that determining the degree of co-localisation of effector and target cells, and the level of peptide expression on targets, are most important for improving estimates of CTL killing rates. Further, while the probabilities of killing upon conjugation of CTL with T and B cell targets are similar, T cells take substantially longer to kill than B cells, an effect that may be important when CTL numbers are limiting.

## Introduction

Cytotoxic T lymphocytes (CTL) prevent the spread of intracellular pathogens through T cell receptor (TCR) recognition of pathogen-derived peptides presented on MHC class I molecules on the surface of infected cells. CTL may have several modes of action but their canonically understood role is to kill cells recognised as infected, either through delivery of lytic mediators through the target cell membrane or engaging ligands on the cell surface that induce apoptosis.

Quantifying the kinetics of CTL killing has been of interest for many years [1]–[19] (see ref. [20] for a review) and is important for at least two reasons. First, knowledge of the rate at which individual CTL can survey and kill cells allows us to derive estimates of the numbers or tissue densities of CTL required to contain an infection. Second, developing tools to measure the kinetics of the different processes involved in lytic activity (locating cells, forming stable conjugates, lysing the infected cell and dissociating from it) may help us to understand how ineffective or exhausted CTL are functionally impaired or to identify bottlenecks in the lytic process that may be potential targets for augmenting CTL responses.

Early studies of CTL-target dynamics were performed almost exclusively *in vitro* but more recently there has been some focus on data from splenic killing assays, using variants and generalizations of the experimental and modeling approach taken by Barchet *et al.* [21] and Regoes *et al.* [11]. There, mice are challenged with a pathogen and following the clearance of infection, a mixture of isogenic splenocytes either pulsed with pathogen-derived peptides or left as unpulsed controls is injected intravenously. Proportions of both populations accumulate in the spleen, where the peptide-pulsed cells can be killed by resident epitope-specific CTL. Models of the kinetics of the transferred cell populations in the spleens in the hours following transfer have yielded estimates of the rate at which single spleen-resident CTL are able to survey and kill.

These models assume that CTL and targets are interacting in a well-mixed environment and have provided reasonable descriptions of the data with the assumption that peptide-pulsed targets are lost with first-order kinetics. When CTL are present in excess – that is, at high effector:target (E:T) ratios – one can assume that the total rate at which targets are killed is not limited by the time each CTL takes to lyse its target [22]. The simplest model of CTL activity then assumes that the *per-capita* rate of loss of targets is 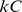, where 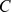 is a measure of CTL density or numbers in the spleen. The units and magnitude of 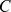 dictate the interpretation of the constant 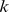, but if 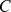 is measured as a proportion of all surveyable cells in the spleen, 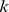 is the rate at which one CTL can move between cells of any type, multiplied by the probability of lysis upon engagement with a peptide-pulsed or infected cell (Text S1, section A). We term 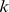 the ‘effective surveillance rate’. If killing is assumed to occur with 100% efficiency, 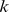 is simply the rate of CTL surveillance, and has been estimated to be in the range 1–35 cells per minute in a variety of experimental infection systems [11], [12], [16], [18]. In simple models of well-mixed CTL and targets, knowledge of this parameter, the time taken for CTL to lyse infected cells, and the uncontrolled pathogen growth rate are sufficient to calculate the critical CTL density required to control an infection [22].

These studies took a rather coarse-grained view of CTL-target dynamics that may mask several potential sources of heterogeneity and CTL biology. First, the assays are performed with mixed splenocyte populations but these models assumed all cells are detected and killed with equal efficiencies. Are some cell populations intrinsically easier to kill than others? Second, although CTL have been shown to be able to respond in a dose-dependent manner to very low levels of peptide-MHC (pMHC) ligands on a cell surface [23], [24], it is not clear whether this susceptibility varies across cell types, or at what stage in the killing process any effect of pMHC availability is manifest. Third, the models assume the spleen to be a single compartment, but it is a heterogeneous environment with areas enriched for T cells, B cells and red blood cells, raising the possibility that cell populations are not well-mixed and cells of different types may be exposed to different CTL densities. To explore these issues we used a combination of *in vivo* and *in vitro* killing assays to examine the influence of target cell heterogeneity, peptide dose and spatial heterogeneity on the kinetics of CTL activity *in vivo*.

## Results

### Basic model of splenic killing kinetics

**Figure 1:**
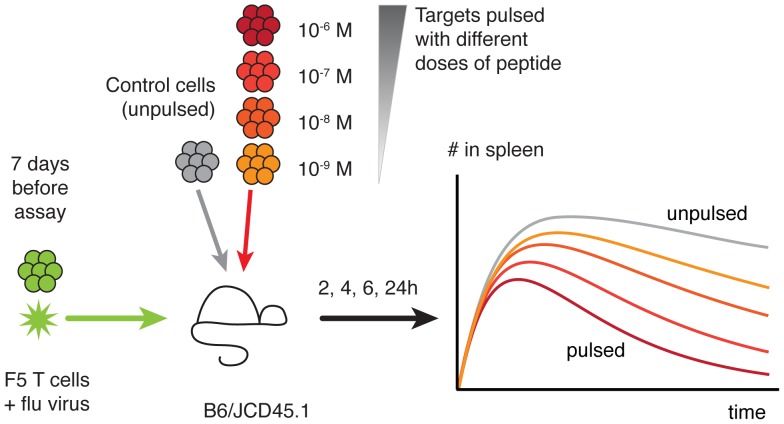
The *in vivo* killing assay.

We revisited the splenic killing assay in the setting of influenza infection (Figure 1). The experimental system is detailed in Materials and Methods, but briefly, TCR transgenic F5 T cells specific for the NP68 influenza epitope were transferred to congenic mice and challenged with systemic administration of live influenza virus. Our calculations depended on being able to enumerate the CTL capable of participating in lysis of pulsed targets. Tetramer staining one week after challenge revealed that transferred F5 cells outnumbered endogenous CTL specific for the NP68 influenza epitope roughly 500-fold in the spleen (Text S1, section B). We therefore assumed that F5 numbers were a reasonable approximation to the number of antigen-specific effector cells in the spleen. To assess the cytotoxic activity of these CTL, total splenocytes from donors were pulsed with four different doses of peptide, with each dose associated with a different level of cell dye, then transferred intravenously to influenza-immunised hosts. T and B cell targets were identified by the expression of either TCR or B220, yielding 8 different target cell populations.

The original model of the kinetics of transferred cells in blood and spleen [11] assumed pulsed and unpulsed cells flow from blood to spleen in the same ratio as they are present in the inoculum, and that transferred, unpulsed cells are not lost from the spleen after entry over the course of the assay. We found direct evidence challenging both of these assumptions (Text S1, section C), so as a starting point we extended the model to allow for (1) a rate of enrichment 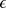 of unpulsed cells relative to pulsed cells in the blood over time, and (2) a rate of loss 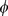 of the transferred cell populations from the spleen through egress and/or death due to non-CTL-related mechanisms. The basic model is then represented with the following:
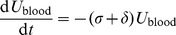
(1)

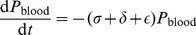
(2)

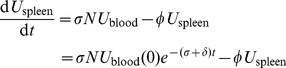
(3)

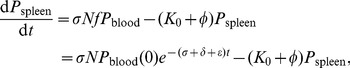
(4)where in equations 3 and 4 we have inserted the solutions for the time-dependent densities of transferred cells in blood (equations 1 and 2). All populations are assumed to enter the spleen from the blood at *per-capita* rate 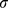. Unpulsed and pulsed cells die in the blood or migrate into locations other than the spleen at rates 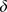 and 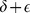, respectively. 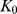 is the *per-capita* rate of loss of pulsed targets in the spleen due to lysis by CTL, and 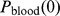 and 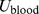
(0) are the initial concentrations of pulsed and unpulsed cells in the blood, respectively.

The quantity of interest is the ‘fractional killing’ in the spleen or the extent of loss of pulsed relative to unpulsed cells, 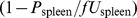 for each transferred cell population, in which the quantity 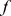 corrects for departures from a 1∶1 ratio of pulsed to unpulsed cells in the inoculum (see Materials and Methods). The fractional killing therefore lies in the range [0,1]. Using this quantity controls to some extent for variation across animals in the number of cells recovered from the spleen. Because it is dimensionless, units of measurement of cell numbers in blood and spleen do not need to be specified in this calculation. The constant 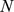 in equations 3 and 4 relates the units of cell numbers in the blood to those in the spleen, and appears linearly in the solutions to equations 1 – 4. In combination with the spleen influx rate 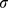 it therefore disappears from the ratio 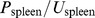 (Text S1, section D).

Before using equations 3 and 4 to estimate the killing rate 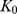 from the fractional killing, the total rate that unpulsed cells leave the blood (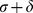) and the splenic loss/egress rate (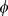) were estimated by fitting equation 3 to the timecourse of unpulsed cells in the spleen (Text S1, section C). We chose to measure cell population sizes as proportions of total splenocytes. This measure exhibited a much smaller coefficient of variation than total numbers in the spleen (Data S1), possibly due to variation in spleen size across animals and associated differences in the total rate of ingress of lymphocytes. The excess rate of loss of pulsed over unpulsed targets from the blood, 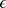, was estimated independently from observations of the ratio of pulsed to unpulsed transferred cells in the blood up to 18 hours post-transfer (Text S1, section C). Using these estimates (shown in Table 1) left only the target cell death rate due to CTL, 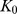, to be estimated from the timecourse of the fractional killing, 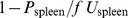. The further assumption of mass-action at the whole spleen level corresponds to 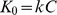 where 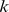 is the effective surveillance rate and 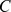 is the measured number of F5 CTL in the spleen as a proportion of total splenocytes.

**Table 1:** Parameter estimates for the models of target cell kinetics in blood and spleen.

Model	Parameter description	Name	Dose	T cell		B cell	
	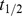 of unpulsed in blood (h)	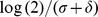	–	1.3	(0.793, 2.21)	1.5	(0.986, 2.42)
	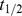 of unpulsed enrichment in blood (h)	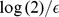	–	4.31	(3.36, 6.02)	11.4	(7.13, 28)
	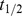 of spleen egress (h)	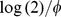	–	13.7	(9.37, 20.1)	21.8	(14, 35.5)
	Pre-killing time lag (h)	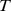	–	1.17	(1.04, 1.32)	1.31	(1.19, 1.45)
**Decay**	Base target half-life (h)	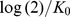	6	0.485	(0.407, 0.578)	1.24	(1.05, 1.45)
			7	0.727	(0.602, 0.875)	1.27	(1.06, 1.51)
			8	0.917	(0.736, 1.14)	1.46	(1.2, 1.76)
			9	2.72	(2.02, 3.67)	2.32	(1.83, 2.93)
	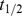 of killing decay (h)	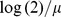	6	2.68	(2.37, 3.03)	8.02	(6.64, 9.71)
			7	2.96	(2.56, 3.44)	6.24	(5.18, 7.5)
			8	2.78	(2.32, 3.32)	5.75	(4.72, 7)
			9	3.49	(2.64, 4.64)	5.07	(3.93, 6.53)
**Hidden-target**	Susceptible target half-life (h)	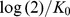	6	0.979	(0.889, 1.07)	1.62	(1.46, 1.79)
			7	1.28	(1.14, 1.42)	1.73	(1.55, 1.93)
			8	1.47	(1.28, 1.68)	1.94	(1.71, 2.19)
			9	2.51	(2.01, 3.14)	2.55	(2.15, 3.03)
	Susceptible fraction	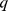	6	0.945	(0.936, 0.953)	0.984	(0.98, 0.987)
			7	0.907	(0.891, 0.92)	0.959	(0.95, 0.967)
			8	0.827	(0.801, 0.85)	0.927	(0.91, 0.94)
			9	0.572	(0.523, 0.62)	0.771	(0.728, 0.808)
**Hybrid**	Susceptible target half-life (h)	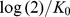	6	0.903	(0.814, 0.996)	1.47	(1.32, 1.64)
			7	1.21	(1.09, 1.35)	1.59	(1.42, 1.79)
			8	1.46	(1.29, 1.66)	1.83	(1.62, 2.07)
			9	2.93	(2.47, 3.48)	2.71	(2.33, 3.17)
	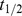 of loss of susceptibility (h)	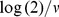	6	40.4	(34.2, 47.9)	179	(144, 223)
			7	26.3	(22.1, 31.3)	73.6	(58.9, 91.7)
			8	14.7	(12.3, 17.7)	42.6	(34.1, 53.5)
			9	7.22	(5.83, 8.96)	15.2	(11.9, 19.5)

**Figure 2:**
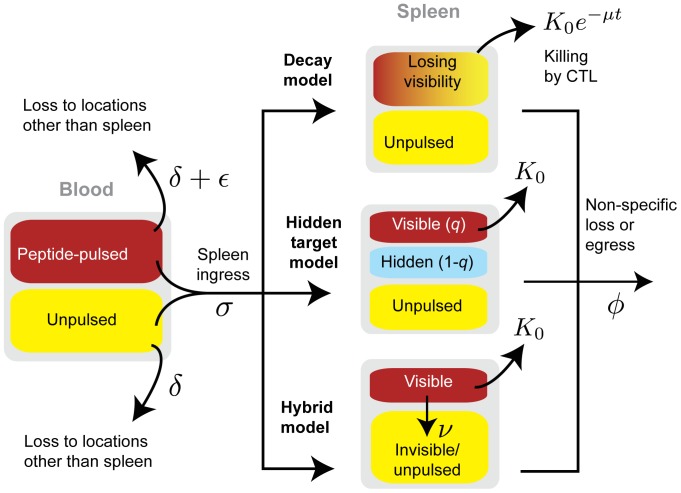
Three alternative models of the kinetics of peptide-pulsed and unpulsed cells in the blood and spleen. In the Decay model, peptide-pulsed targets uniformly and gradually lose susceptibility to lysis; in the Hidden-target model, only a proportion 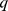 of peptide-pulsed targets are susceptible to lysis; and in the Hybrid model, peptide-pulsed targets maintain susceptibility but transition at *per-capita* rate 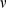 into a non-susceptible state.

Equations 1-4 predict that the fractional killing should asymptotically approach 1, simply because the *per-capita* rates of loss of all populations are constant over time and the loss rate is higher for pulsed than unpulsed cells. However the loss of pulsed targets stopped short of 100%, an effect more pronounced for T than B cells and increasingly apparent at lower peptide doses. To account for this saturation, we explored three extensions of the basic model, illustrated in Figure 2 and detailed in Text S1, section D.

#### 1. The decay model

Here we assumed that the CTL-mediated death rate of pulsed targets in the spleen falls exponentially with time [12], . Such a decay might reflect the exhaustion of effector CTL required to kill multiple times, although in our assays the ratio of CTL to pulsed targets was 3 or greater (Text S1, section E) and so this mechanism seems unlikely. Another interpretation of the decay over time is that peptide-pulsed cells became progressively less visible to CTL due to loss of peptide from, or natural turnover of, MHC on the cell surface. This will reduce the effective surveillance rate. In this model the CTL-mediated death rate decays as 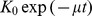.

#### 2. The hidden-target model

Here we assumed that only a proportion 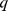 of the population is susceptible to killing. The remaining fraction 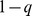 might represent a proportion of pulsed targets being physically inaccessible to CTL or refractory to killing over the entire course of the assay.

#### 3. The hybrid model

Here pulsed targets transition at some rate 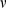 into a non-susceptible population, a model similar to that proposed in ref. [13] and reflecting elements of both the decay and hidden-target models. This model might represent a loss of peptide from target cells, which renders them invisible to CTL below some threshold level of surface expression of pMHC.

### Modeling blood-spleen recirculation

**Figure 3:**
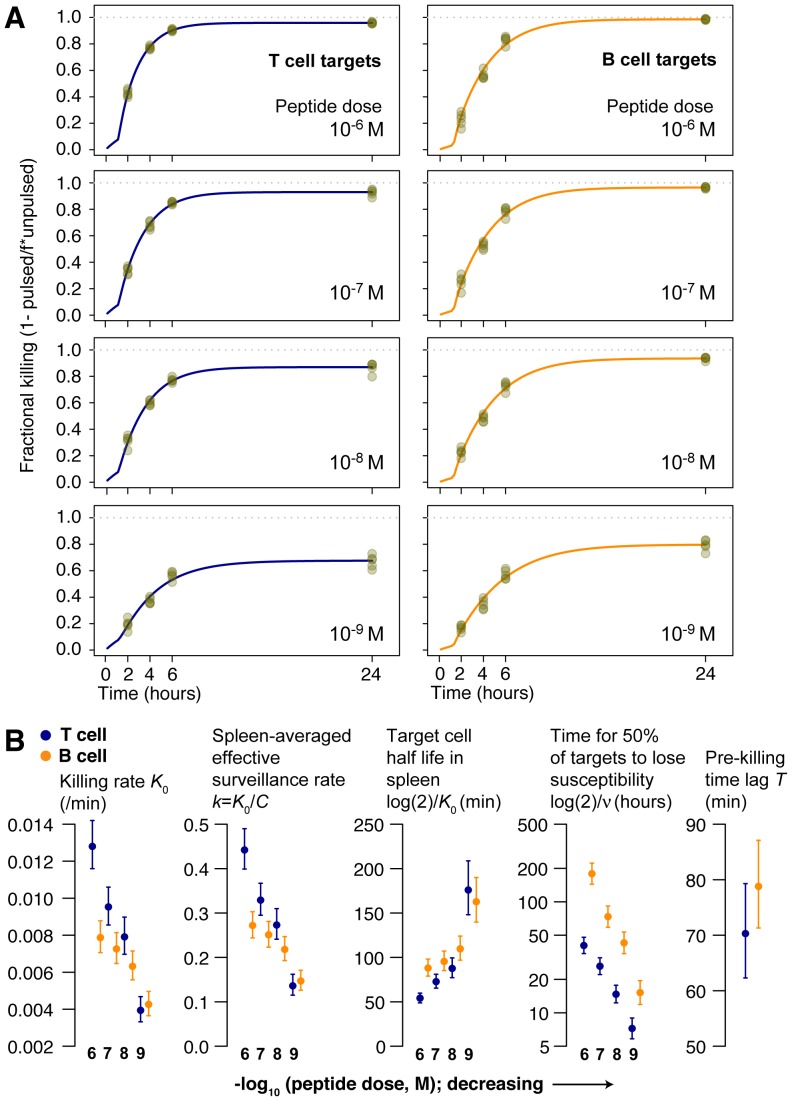
Modeling the in-vivo cytotoxicity assay. **A**: Timecourses of fractional killing in the spleen, broken down by peptide dose between 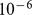 M and 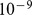 M, decreasing top to bottom) and target cell type, with 5 mice per time point (green points). Curves show the predictions of the best-fitting (hybrid) model. **B**: Key best-fit parameters governing killing of pulsed targets in the spleen, with 95% confidence intervals.

We combine egress and non-specific cell death in the spleen in a single loss rate 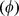. Similarly the rate 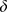 combines both death in the blood and the net rate of extra-splenic sequestration of unpulsed targets. An integrated model of spleen-blood kinetics would allow for a proportion of targets lost from the spleen to re-enter the circulation, a process that would contribute to the enrichment for unpulsed cells in the blood over time. Instead we represent the population dynamics of cells in the blood semi-empirically with equations 1 and 2, which recapitulate the kinetics of the pulsed:unpulsed ratio in blood and of unpulsed cells in the spleen (net influx slowing to zero by 6h, followed by a net loss). Killing also slows to near zero by 6h (Figure 3A) and our estimates of parameters related to CTL activity are insensitive to the splenic loss rate 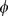, suggesting it is reasonable to use our description of blood kinetics. This assumption also eliminates the need to estimate the potentially different non-specific death rates in blood and spleen.

### CTL killing rates vary with target cell type and peptide dose

We fitted these three models to the data, estimating the CTL-mediated killing rate 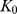 and the additional parameter for each model (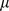, 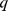 or 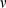) separately for the two target cell populations. To complete the analysis we found that the addition of a third parameter, a time lag 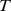, was required to account for the fact that the fractional killing curves did not extrapolate cleanly to zero at the beginning of the assay (Figure 3A). 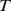 represents a delay between transfer and the first evidence of death of pulsed cells. It might comprise the mean time taken to migrate from blood into the spleen and across the marginal zone into white pulp where we would expect specific CTL to be resident in the greatest numbers. It may also comprise the handling time, the time taken for a target to be killed following conjugation with a CTL. Because splenic effector:target ratios were high in these assays (Text S1, section E), few CTL are expected to kill more than once and so the handling time is not expected to limit the total rate of killing, but it may act to delay the appearance of the first apoptotic cells. If the times to complete any or all of these process are non-exponentially distributed, or if more than one process is operating, there may be a discernable delay before killing is observed. We model the effect of migration and/or handling time by setting the killing rate 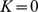 for times 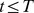 and 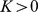 for 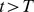, where 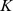 is time-dependent for the decay model, 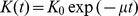, and a constant 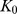 for the hidden-target and hybrid models. We found that our estimates of the time lag 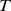 did not vary significantly across peptide doses and so assumed that it took values specific to the T or B cell populations only.

Due to the non-nested nature of the models we compared their abilities to describe the data using the Akaike Information Criterion (AIC) [25]. Since the three models contained equal numbers of parameters, the AIC could be calculated simply as *n* log(RSS) where RSS is the residual sum-of-squares and 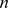 is the number of datapoints. This quantity is the negative of twice the (maximum) log-likelihood up to an additive constant in 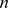 which disappears when comparing models. We found strongest support for the hybrid model and the weakest for the decay model, for both T cell and B cell targets (Hidden-target vs. hybrid model, 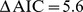 for T cells, 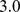 for B cells; Decay vs hybrid model, 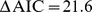 for T cells, 17 for B cells). The interpretation of these differences is that the relative probability of the hidden-target and not the hybrid model minimising the information lost in describing the data is 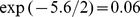 for T cells and 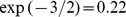 for B cells. However the fits were visually indistinguishable on both the absolute scale and the logit-transformed scale on which fitting was performed, and parameter estimates were comparable. Graphs of fits and parameter estimates for the Hybrid model are shown in Figure 3 and parameter estimates for all three models are shown in Table 1. Reduced models with no pre-killing lag time 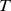 yielded substantially inferior fits (for the hybrid model, 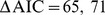 for B and T cells respectively).

Our key observations are that every model indicated that within the susceptible populations B cells were killed significantly more slowly *per-capita* than T cells at all doses except the lowest, and for both populations the rate of killing of susceptible targets 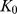 fell with peptide dose (Figure 3B and Table 1). While we will argue that spleen-averaged estimates may be misleading, to allow comparison with estimates of the effective surveillance rate 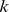 from other studies and pathogens, we quote here the spleen-averaged 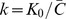 where 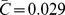 was the average number of F5 cells as a proportion of splenocytes across all animals and all timepoints. The effective surveillance rates of F5 effectors for T cell targets (with 95% CIs) ranged from 0.14 (0.12–0.16)/min at the lowest peptide dose to 0.44 (0.40–0.50)/min at the highest dose, and from 0.15 (0.13–0.17)/min to 0.27 (0.24–0.30)/min at low/high doses for B cells. Interpreting these figures in terms of numbers of targets surveyed per unit time is complicated by the fact that 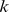 combines both the base cell-cell surveillance rate 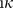 and the probability that a CTL kills a pulsed target on encounter, 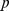, which may be 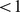; so the estimates of 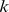 at higher peptide doses where one might expect killing efficiency to be greatest [26] are likely closest to the base surveillance rate 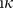 but still a lower bound. Mempel *et al.* [27] used intravital imaging to study the lysis of peptide-pulsed B cell targets by specific CTL in a popliteal lymph node, and estimated that CTL take 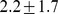 minutes *in vivo* to survey cells not expressing cognate antigen. Our figures are consistent with this if one assumes that in their system the time for a CTL to migrate between surveyable cells was short.

Estimating 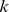 from the target cell death rate 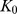 also requires the assumption that all target cell populations are exposed to the same spleen-averaged density 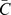. This assumption is questionable, as we see below. Another caveat is that 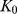 is the rate of killing of susceptible targets only; if any targets are refractory or inaccessible to CTL, then models that assume all targets are susceptible such as in [11] will underestimate 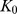. Estimates of 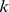 derived from these models may then also be lower bounds. Nevertheless the effective surveillance rates in the setting of influenza infection and F5 transgenic CTL are slower than those estimated using LCMV or polyomavirus infections [11], [12], [16], [18], an issue we return to in the Discussion.

### Splenic egress and differential loss of pulsed and unpulsed targets from blood influence estimates of effective surveillance rates only weakly

Modeling splenic egress and enrichment of unpulsed targets in the blood was required to explain the kinetics of unpulsed targets in the spleen and of the pulsed:unpulsed ratio in the blood, respectively (Text S1, section C). Including these processes in the models of killing improved their qualities of fit slightly (for the hybrid model, neglecting splenic egress and blood enrichment increased the AIC by 1.6 for B cells and 1.9 for T cells) but had no substantial impact on parameter estimates or the relative support for the three models. This is likely due in part to a disparity in timescales. Our estimates of the time for the pulsed:unpulsed ratio to halve in the blood were 4.3 h for T cells and 11.4 h for B cells (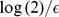, Table 1). In contrast, the total rate of influx of unpulsed cells into the spleen is proportional to 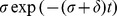 in these models and was estimated to halve in 1.3 h for T cells and 1.5 h for B cells (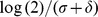, Table 1). Thus, the differential influx of the pulsed and unpulsed cells into the spleen became apparent only after the majority of targets had accumulated. Further, we assumed that the splenic egress or non-specific loss rate 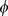 applied to pulsed and unpulsed populations equally, and so including this process only weakly influences the timecourse of the pulsed:unpulsed ratio in the spleen. We note that other estimates of times to transit the spleen are lower, and that T cells may egress more rapidly than B cells [28], [29], suggesting that killing in the spleen may indeed contribute to the enrichment for unpulsed cells in the blood.

The delay before killing, 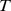, was comparable for T and B cells at approximately 75 minutes. T and B cells take 20–30 minutes following intravenous transfer to cross the marginal zone of the spleen and enter the white pulp [30]. A handling time between encounter and breakup of the target cell has been observed in many studies. A study of killing of B cells *in vivo* found CTL took between 9–25 minutes to lyse targets after conjugation [27]. Note that the models predicted an increase in the fractional killing in the spleen during the lag period 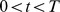, most strongly for T cells (Figure 3A and Table 1). However by neglecting recirculation we predict this is due to the slow but significant enrichent for unpulsed over pulsed T cell targets from the blood into other anatomical locations, and not killing in the spleen itself.

### No detectable signature of mass-action killing kinetics at the whole-spleen level

To investigate whether we could detect evidence of mass-action killing operating, following ref. [12] we allowed for the base killing rate 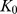 to be specific to each mouse, 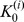, and assumed it was linearly proportional to the density of effector CTL in the spleen of that animal, 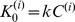. Here 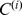 is the measured number of F5 CTL as a proportion of all splenocytes, and 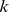 is the effective surveillance rate. We assumed that 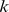 was constant across animals but took values specific to each target cell type (T or B cell) and peptide dose.

Effector:target ratios were greater than 3 in these assays (Text S1, section E), so if CTL and targets were well-mixed in the spleen and moving randomly, mass-action might be expected to hold. However, we found that across all peptide doses and target cell types the models using a simple population-average killing rate 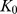 described the data significantly better than those with mouse-specific, mass-action killing rates 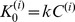. Indeed we saw no significant positive correlation between fractional killing and F5 CTL, either in total numbers or as a fraction of splenocytes, at any peptide dose or timepoint, after correcting for multiple comparisons (Text S1, section F). We saw roughly 40% variability across animals in the total number of F5 CTL recovered from the spleens, 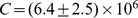. However the dependence of the rate of loss of targets on the size of the CTL population might be expected to be an increasing function of their density, rather than of total numbers alone; the rate of encounter of targets with a given number of randomly dispersed CTL would be expected to vary with spleen volume if the populations are well-mixed. F5 CTL numbers as a proportion of all splenocytes were highly consistent across animals and timepoints (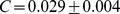, or 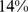 variation). We conclude that our data do not provide sufficient power to support or refute the mass-action hypothesis at the whole-spleen level, nor do they allow us to quantify any functional dependence of killing rates on CTL densities. Ganusov *et al.* [19] performed these assays in the context of LCMV infection, using adoptive transfer of specific CTL and varying their number over three orders of magnitude, and did indeed find evidence for a linear dependence of splenocyte death rates on CTL numbers or frequencies, and so in the analyses below we retain the possibility that mass-action operates.

### Arrest in killing likely arises from a combination of progressive loss of peptide from the cell surface and heterogeneous uptake of peptide, but not spatial effects or MHC turnover

Another key observation derived from the hybrid model is that the average rate at which pulsed target cells lost susceptibility increased by an order of magnitude as peptide dose fell from 10^−6^ M to 10^−9^ M, most rapidly for T cells. This effect was reflected in the hidden-target model by the susceptible fraction declining with dose, and generally being smaller for T cells than B cells (Figure 3B and Table 1). These dose-dependencies suggest that heterogeneity in susceptibility derives from properties intrinsic to target cells rather than global effects such as cells migrating into regions in the spleen inaccessible to CTL.

In the light of these observations, the following is a mechanism of loss of susceptibility compatible with the hybrid model:

- Peptide is progressively shed from targets or internalised;
- CTL are able to recognise targets with a probability that is an increasing function of peptide dose, likely sigmoid;
- Targets become effectively invisible below a threshold level of surface expression of cognate pMHC.

Peptide-pulsed targets would be expected to exhibit a unimodal distribution of pMHC densities at each dose, and if peptide is lost this distribution will shift towards lower pMHC densities with time. The lower the dose of peptide, the closer to any threshold of detection the initial distribution will be, and so the average rate of loss of susceptibility across the entire target population (the rate 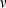 in the hybrid model) will increase, as observed.

**Figure 4:**
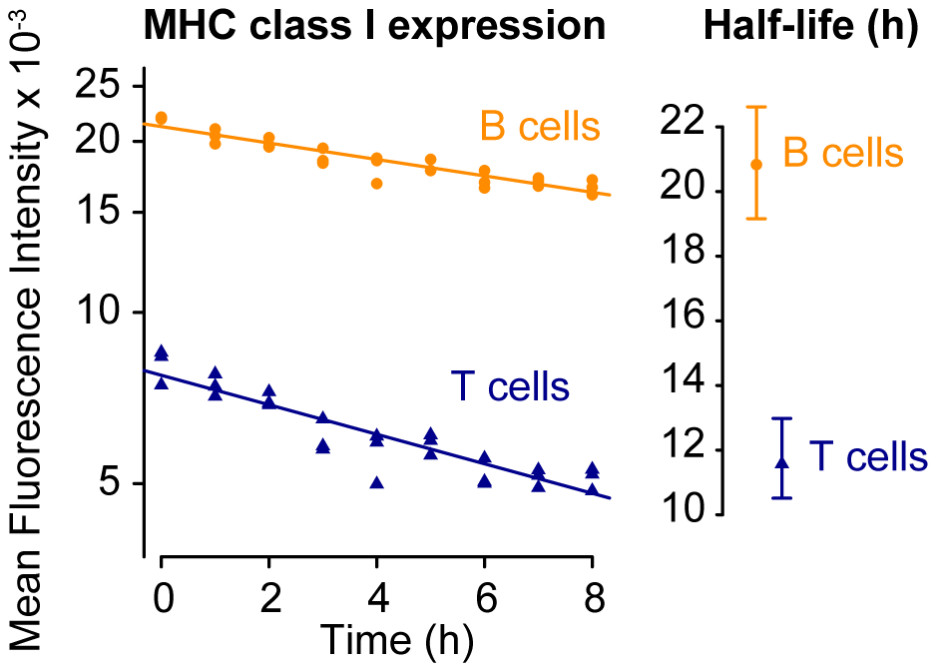
Kinetics of MHC class I turnover on B and T cells. MHC class I expression was measured (MFI, arbitrary units) following blocking of its transport to the cell surface. Its decay followed approximately first-order kinetics, and occurred faster on T than on B cells.

To explore the peptide-loss hypothesis, we quantified the expression and turnover of MHC class I on T and B cells *in vitro* by blocking transport of MHC class I to the cell surface and observing the kinetics of its internalisation (see Materials and Methods). We found T cells expressed MHC class I on the cell surface at 2–3 fold lower levels than B cells, and MHC was lost approximately twice as rapidly (Figure 4; half-lives of approximately 11 and 21 h for T and B cells respectively). If these peptide-MHC turnover rates *in vitro* reflect the rate of turnover *in vivo*, then in our splenic cytotoxicity assays pMHC densities on the T cell targets fall only approximately 2-fold before the bulk of surviving pulsed cells are below a threshold of detection and killing has stopped. This means that for loss of MHC to underlie arrest in the hybrid model, peptide doses covering four orders of magnitude result in four target cell populations clustered closely in pMHC expression just above the limit of detection. Parsimony then suggests that it is unlikely that MHC internalisation alone explains the arrest of killing, as suggested previously [13].

MHC turnover imposes only a lower limit on the rate of loss of visibility of targets, as peptide may dissociate from the MHC class I to which it is bound. While we do not have an estimate of the lifetime of the NP68/H-2Db complex used in our assays, peptide-MHC class I dissociation half-lives of 1–4 h have been reported in other systems [31]–[33] and so loss of peptide from targets remains a potential explanation for loss of susceptibility. However we cannot exclude a contribution from heterogeneity in peptide uptake by targets such that at decreasing peptide doses, an increasing proportion of pulsed targets are already below a threshold of detectability by CTL at the beginning of the assay. The hidden-target model, which describes the data with fidelity comparable to the hybrid model, at least visually if not statistically, is an expression of this heterogeneity in initial conditions.

Finally, the dependence of the B cell killing rate on peptide dose was weaker than that of T cells (Figure 3B and Table 1). One explanation for this is that the higher level of MHC expression on B cells means at higher peptide doses the population lies more completely within a saturating region of the curve relating dose to susceptibility to CTL.

### Differences in rates of killing of T and B cell targets cannot be explained purely by differences in CTL densities in T and B cell areas using a mass-action model

**Figure 5:**
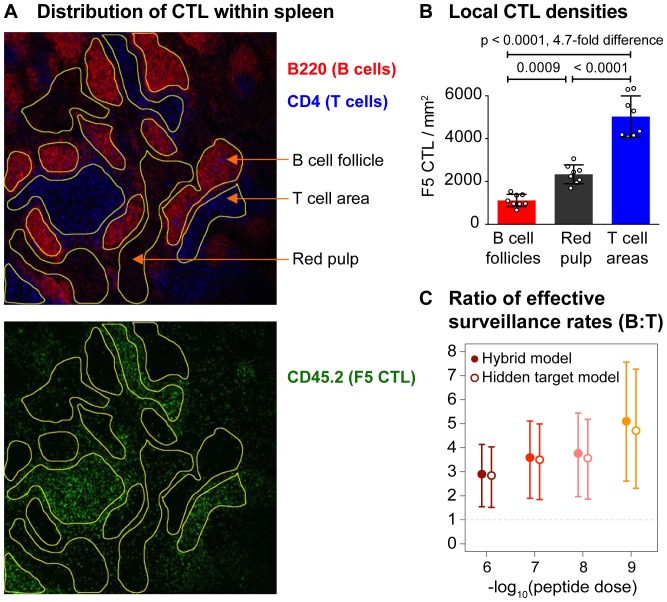
Influenza-specific F5 CTL are distributed heterogeneously within the spleen. **A**: Imaging of the spatial distribution of F5 CTL within T and B cell areas and red pulp of spleen. **B**: Statistical comparison of F5 densities in these areas. **C**: Using equation 5 with these data to establish the relative efficiency with which F5 CTL kill B and T cell targets after locating them.

All three models indicated that the *per-capita* rate of killing by CTL was lower for B cell targets than for T cells. This may be because B cells are intrinsically less susceptible to lysis at a given peptide dose, that B and T cells encountered different local densities of CTL, or that the CTL were less motile in B cell areas than in T cell areas. To begin to discriminate between these (non-exclusive) possibilities, we used microscopy to quantify the distribution of effector CTL within the spleen (Figure 5A). F5 CTL were indeed distributed heterogeneously, with the majority (60%) in T cell areas, roughly 4-fold fewer (13%) in B cell follicles, and more than a quarter (27%) in red pulp (Figure 5B).

Since at least a proportion of the intravenously injected T and B cell targets may migrate to their respective areas in the spleen, it then seems likely that the different target cell populations are exposed to different local densities of CTL. To further explore the relative contributions of this spatial heterogeneity and potential differences in susceptibility to lysis, we used this spatial data together with the T and B cell death rates to estimate the relative ability of F5 CTL to kill peptide-pulsed B and T cells. To separate the effects of CTL numbers and susceptibility we began by assuming that mass-action held within the T and B cell areas and killing of each population was restricted to its relevant area. Targets and CTL might reasonably be assumed to be randomly distributed within each.

In the hybrid and hidden-target models the *per-capita* rate of killing of the susceptible population of peptide-pulsed T cells in the spleen is 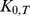. If these T cells are restricted to T cell areas and are exposed to specific CTL at local density 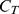, this killing rate must be equal to 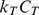 where 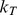 is the effective surveillance rate in T cell areas. So 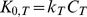, and similarly for B cells. Note that this calculation does not depend on the relative volumes of B and T cell regions in the spleen, which we estimated to be 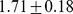 (mean 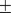 s.e.m.) by raising the ratio of their areas in the imaged sections to the power 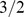. Different degrees of crowding of T and B cell targets in the spleen are represented by different values of the parameter 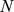, which relates density units in blood and spleen but disappears from the estimation of 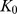 for each population. Then
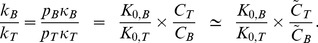
(5)Here 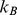 and 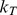 are decomposed into the probabilities of lysis following encounter with a CTL, 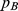 and 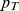, and the base CTL surveillance rates in B and T cell areas, 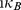 and 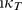.

In this calculation we have replaced the local densities of CTL in each of the T and B cell areas (as fractions of total splenocytes within each region) with 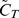 and 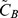, the measured local CTL densities in units of cells per 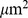 of spleen section. Because these sections were of the order one cell width deep, the CTL densities measured per unit volume are then approximately these cell numbers per unit area divided by the section depth. We assume the densities of total surveyable cells are the same in T and B cell areas, and so 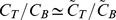. Using the target cell death rates derived from the hybrid model, we estimate that on a per-CTL basis B cells are killed 3-5 times more rapidly than T cell targets, with the lower boundary of the 95% confidence interval lying above 1, at all peptide doses (Figure 5C, solid circles). The hidden target model yielded very similar estimates (Figure 5C, open circles).

In summary, under the assumptions of mass-action and restriction of the populations to their respective areas in the spleen, the difference in local densities of CTL was too large to explain the difference in killing rates of T and B cells pulsed with the same dose of peptide, and so the relative paucity of CTL in B cell areas is compensated to a degree by a higher effective surveillance rate (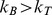).

### Analysis of CTL-target dynamics *in vitro*

This difference in 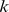 might stem from susceptibility to lysis; for instance, the 2–3 fold difference in MHC expression by B cells might contribute to a higher probability of detection by CTL upon encounter, 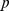, at a given peptide dose. It might also arise from differences in the motility of CTL within T and B cell areas, 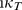 and 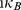. To narrow down the possibilities even further, we wanted to estimate the intrinsic susceptibilities of T and B cells to lysis by CTL and assess their dependence on peptide dose, while minimising any effects of spatial heterogeneity or CTL motility.

Lysis is a multi-stage process. The CTL must encounter and survey the cell, detect that it bears the relevant peptide, form a stable conjugate, initiate lysis and eventually disengage from the apoptotic cell. We wanted to identify at which stage(s) of the killing process any differences between T and B cells or across peptide doses were manifest most strongly. To do this we performed an *in vitro* cytotoxicity assay using T and B cell targets pulsed as before with different doses of the peptide, and co-localised with F5 CTL activated *in vitro* (see Materials and Methods). These populations were co-cultured for between 0 and 120 minutes, allowing us to follow the kinetics of free targets (*S*), the number of CTL-target conjugates in which the CTL had not degranulated (stained negative for LAMP1a at the cell surface, 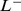), and the number of conjugates in which the CTL had degranulated (LAMP1a detected at the cell surface, 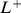), assumed to indicate lysis.

**Figure 6:**
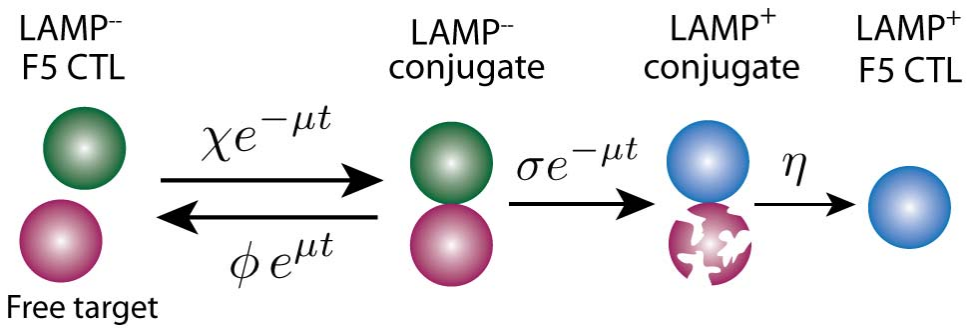
Schematic of model of killing of peptide-pulsed targets by specific CTL *in vitro*. CTL that have not previously engaged in lysis (LAMP^+^, green) initially form conjugates with peptide pulsed targets (purple) with mass-action kinetics at rate 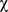. These dissociate at rate 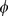 or remain stable till the CTL shows signs of delivery of lytic granules (LAMP^+^. The CTL stays bound to its (presumed) apoptotic targets for mean time 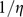 and then dissociates as in the LAMP^+^ state (blue).

The following generalisation of the hybrid or decay models described the kinetics of these populations well (Figure 6):
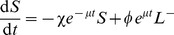
(6)

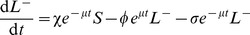
(7)

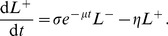
(8)


The term proportional to 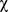 is the total rate of formation of conjugates between targets and LAMP1a^−^ CTL. At the beginning of the assay the expected time for a given target to become conjugated is 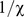. 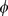 is the initial rate at which a conjugate dissociates without lysis; 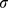 is the initial rate at which a CTL in a conjugate becomes LAMP1a^+^ through degranulation; and 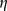 is the rate at which a LAMP1a^+^ conjugate dissociates. CTL were in excess in this assay and so we assumed LAMP1a^+^ cells did not kill again. With this assumption, LAMP1a^+^ conjugates arose directly from LAMP1a^−^ conjugates only.

**Figure 7:**
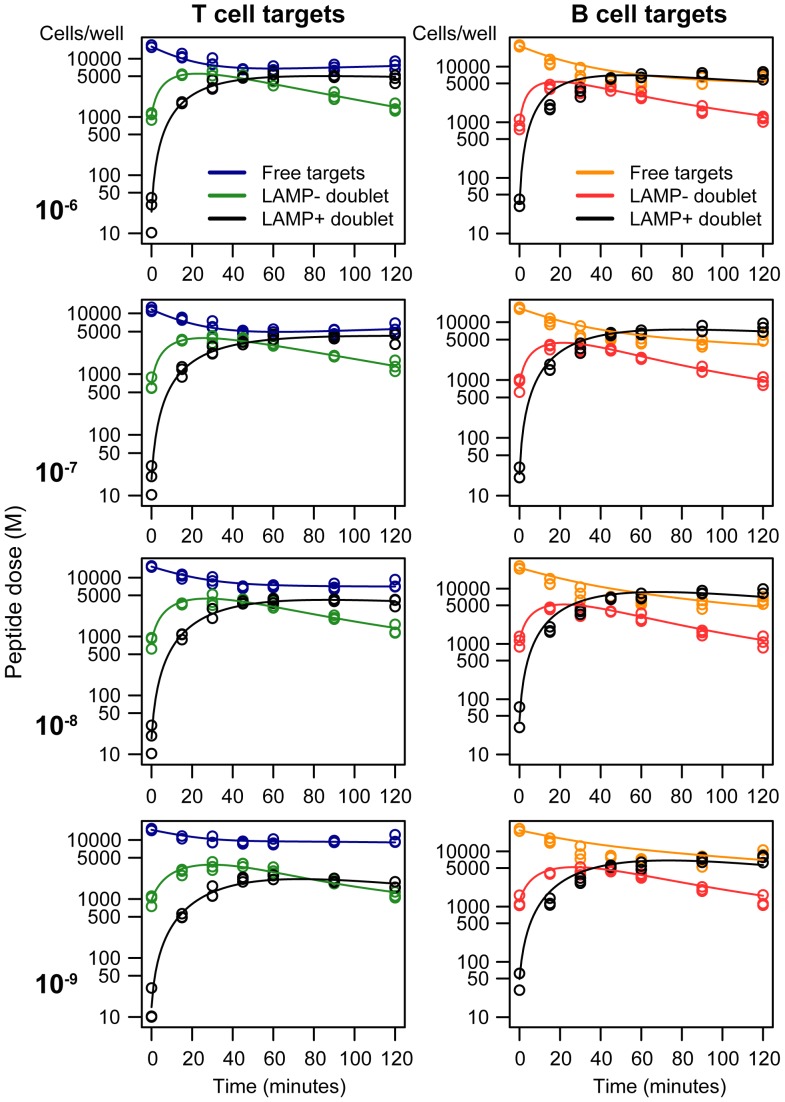
Model fits to data from the *in vitro* killing assay. We applied the model of CTL-target conjugation and killing to data from the *in vitro* killing assay, using T and B cell targets pulsed at four different doses of peptide (decreasing, top to bottom). Free target cells are in blue (T cell) or orange (B cells); green (T cell) and red (B cell) lines are targets in conjugates with F5 CTL that have not degranulated and stain LAMP1a^−^; black lines are LAMP1a^+^ conjugates, which are CTL conjugated to targets following release of lytic granules.

Inspection of the data revealed that the formation of conjugates and killing slowed considerably over the course of the assay, appearing to stop completely after roughly an hour (Figure 7). This was much earlier than the timescale of arrest of killing *in vivo* (Figure 3A), and seems unlikely to be the result of loss of peptide-MHC from the target cells. We observed a roughly 50% loss of effector CTL numbers over the 2h timecourse, accounting for both free CTL and those in conjugates. We speculate that as well as dying, the CTL became increasingly functionally impaired, perhaps related to the release of cytotoxic factors into the culture medium. To capture this behaviour we allowed rate constants to change with time such intially they reflect the interactions between targets and fully-functional CTL, but by 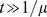, conjugate formation had stopped, and the efficiency of progression to degranulation was zero;
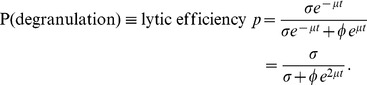
(9)


**Figure 8:**
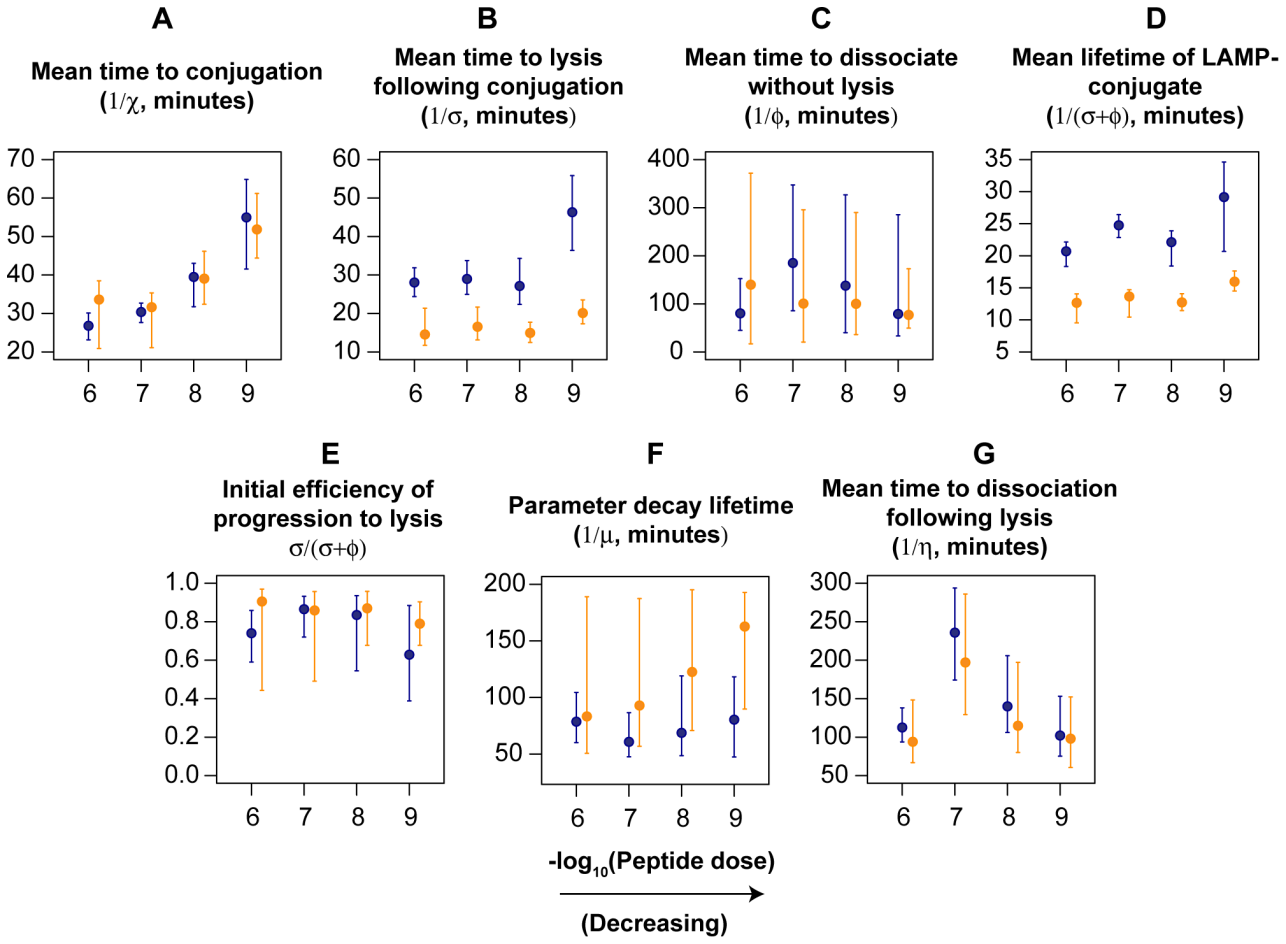
Parameter estimates for the model of CTL-target dynamics *in vitro*. Estimates for T cell targets are in blue; B cells in orange. Vertical bars show bootstrapped 95% confidence intervals derived from resampling residuals 1000 times. See text for the interpretation of the parameter values shown in panels A–G.

Parameter estimates are shown in Figure 8 and rate constants are quoted as their inverses (timescales, in minutes). CTL and targets formed conjugates at similar rates (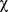) for T and B cells at each peptide dose, but conjugates were slower to form at lower doses (Figure 8A). CTL-B cell conjugates progressed to degranulation (LAMP1a^+^, which we assumed led to lysis) after roughly 15 minutes, independent of dose, while the mean time to degranulation for CTL-T cell conjugates was roughly 30 minutes slowing to 45 minutes at the lowest peptide dose (Figure 8B). We saw considerable uncertainty in the rate of dissociation without lysis, the failure rate 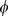 (Figure 8C), but this process was relatively slow and the mean lifetime of LAMP1a^−^ conjugates 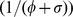 was determined largely by the time to degranulation (Figure 8D). This led to high (50–90%) efficiencies of lysis, 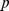, at the beginning of the assay 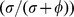, but uncertainty in 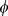 obscured any potential variation in efficiency with peptide dose (Figure 8E). Similarly we detected no significant differences in the rate of change of parameters, 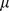 indicating that a progressive loss of CTL functionality affected the killing of all cell populations equally (Figure 8F). Lastly, we estimated that degranulated (LAMP1a^+^) conjugates took between 100 and 200 minutes either to break up or for the target cell to disintegrate (Figure 8G), again with no significant T-B differences.

Multiple CTL bound to single targets may shorten the time taken to kill [4]. We saw evidence for formation of conjugates comprising more than two cells at approximately 10% of doublet numbers after one hour, and stable over time (Text S1, section G). The data were not sufficient to parameterise the dynamics of theses multiples. However, one could reasonably assume that triplets form by a second CTL joining a CTL-target doublet, particularly because CTL were in excess (Text S1, section G). The estimated pre-lytic doublet loss rate 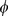 will then comprise both breakup into singlets and formation of LAMP1a^−^ triplets, then quadruplets, etc., within which targets may or may not have an increased probability of being killed. Therefore, by neglecting multiples we may overestimate the true conjugate breakup rate 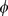, and so our efficiency of killing is a lower bound, with an error of 10% or less. Again, CTL are in excess in these assays and so estimates of the doublet conjugate formation rate 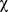 and the doublet lysis rate 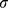 will be unchanged.

In summary, we found that while the rate of conjugate formation fell with peptide dose, there were no detectable differences in the ability of CTL to conjugate with T or B cells; and while T cells subsequently progressed to lysis more slowly than B cells, there were no detectable differences in the efficiency of lysis across cell type or peptide dose. A similar conclusion regarding the effect of peptide dose on conjugation was reached by Jenkins *et al.* [34], who measured the impact of the avidity of TCR-pMHC interations on lysis using transgenic OT-I CTL specific for the OVA_257–264_ peptide. There, the avidity of TCR interactions, assumed positively correlated with peptide dose, impacted the rate of formation of conjugates but had no significant effect on the proportion of conjugates exhibiting clustering of tyrosine kinases at the contact site, an early indicator of TCR signaling and progression to lysis. In contrast, lytic efficiency was found to vary with dose in an *in vitro* tissue model of killing of HIV-derived peptide-pulsed targets [26]. Measurements of rates of conjugate formation and lytic efficiency are somewhat definition-dependent and correlated, however. We may be overestimating lytic efficiency and underestimating the rate of conjugate formation since what we define as a conjugate has remained stable for long enough to be detected by flow cytometry.

## Discussion

### Differences in rates of killing of T and B cells by spleen-resident CTL are likely due to spatial heterogeneity in CTL motility or differences in target cell trafficking patterns, and not intrinsic differences in susceptibility to lysis

The *in vivo* killing assay and the imaging indicated that if killing of T and B cells was restricted to their respective areas in the spleen and rates were locally linear in CTL numbers, differences in local CTL densities were too great to explain the differences in killing rates of the two populations. This suggested that the effect of excess CTL in T cell areas may be partly compensated by more efficient CTL surveillance in B cell areas – either by an increased rate of encounter with cells of all types, or by B cells being killed with a higher probability than T cells following conjugation (Figure 5C). However the rate of formation of conjugates with CTL, and the probability of progression to lysis, were indistinguishable for T and B cells *in vitro*, and so together these assumptions and observations prompt the conclusion that the rate at which CTL survey cells of any type is higher in B cell follicles than in T cell areas.

While that remains to be tested, the assumption of killing of each population being entirely restricted to their respective areas in the white pulp is questionable. Both target cell populations enter the spleen through the circulation and enter the white pulp via the marginal zone. At least a proportion of B cells then migrate through CTL-rich T cell areas *en route* to B cell follicles [30]. Lymphocytes egressing from the spleen do so by transiting the red pulp [29], where more than a quarter of splenic F5 CTL resided in our assay (Figure 5B). Bajénoff *et al.* [30] observed that between 3 h and 8 h after intravenous transfer of isolated splenocytes, B cells were continuing to accumulate in the white pulp from the marginal zone that separates the red and white pulp, roughly a third were resident in B cell follicles, and the remainder were co-localised with T cells. If similar migration patterns and kinetics apply in our assays, the difference in the average CTL densities encountered by the two target cell populations over the assay may be smaller than that inferred simply from the CTL densities in T cell areas and B cell follicles alone. It is possible that this effect alone may account for the differences in T-B killing rates. It is also not critically dependent on the mass-action assumption, requiring only that death rates are increasing functions of CTL density over the conditions found here.

### Differences in handling times across target cell type may only be manifest at low E:T ratios

While we saw no significant differences in the ability of CTL to conjugate with T or B cells in vitro, or in the probability of conjugation resulting in lysis, CTL-T cell pairs took more than twice as long to either break up or progress to lysis. This difference in handling time will not affect the ability of CTL to control an infection when they are in excess, but will become important at lower E:T ratios when an increasing proportion of CTL will be sequestered in conjugates at any time, and so may become limiting [22]. This difference may make growing populations of infected T cells intrinsically more difficult to control than B cells, in the absence of spatial or peptide-dose effects. Note here we are referring to the rate of progression to lysis following encounter, or single-cell behaviour. This is distinct from the population-level killing rate 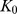 which depends only the rate at which CTL can encounter and identify targets, and not on the handling time, when CTL are in excess or the mean time to locate the next pulsed target us much longer than the handling time.

### Kinetics of CTL killing are pathogen- and context-dependent

We predicted a delay of more than an hour before killing of targets within the spleen was evident. However in an LCMV infection model, Barber et al. [35] observed substantial loss of peptide-pulsed cells in the spleen within 15 minutes, relative to target numbers in uninfected control animals. A similarly rapid decline of pulsed relative to unpulsed targets was observed in a polyoma virus infection model [16], [36]. It is possible that this faster loss derives in part from the systemic nature of those infections, which might lead to greater extra-splenic sequestration or killing of pulsed targets in rapidly perfused organs such as liver and lung. Indeed in an LCMV model, Graw *et al.* [18] saw a roughly four-fold enrichment for unpulsed transferred cells in the blood by 4 hours, compared to the two-fold enrichment in our assay (Text S1, section C). However Barber et al. [35] saw that the early loss of pulsed targets in the spleen was attenuated in mice with CTL lacking Perforin, a membrane pore-forming protein involved in the delivery of cytolytic molecules to the target cell. We might expect filtering of pulsed targets from the blood by 15 minutes to be similar in these and WT mice, since it is initially TCR- and not Perforin-dependent. This strongly suggests that lysis was indeed occurring in the spleen within 15 minutes of cell transfer.

Estimates of the time CTL take to kill targets have varied widely across systems, from minutes [26], [27] to hours [37], and so a discrepancy of this magnitude is perhaps not surprising. F5 CTL may simply take longer to kill; we found mean handling times of 30 minutes or longer for T cell targets *in vitro*, although handling times with B cell targets were shorter (Figure 8B). The longer delay before killing is apparent may also derive from the time taken for CTL and targets to encounter each other. By day 7 the influenza infection is well controlled and so levels of inflammation are likely lower than in the LCMV system, which we speculate may result in reduced CTL motility; and the spatial distribution of specific CTL that we found in our system may differ from those in LCMV infection models [38], which might result in differences in the mean time for ingressing targets to enter CTL-rich areas of the spleen.

### Thresholds of CTL detectability likely lie at very low pMHC densities and peptide uptake is probably heterogeneous

Our results recapitulate previous findings that peptide dose influences susceptibility to lysis by CTL (see, for example, [16], [24], [34]). Threshold effects have also been observed. Purbhoo *et al.* [24] demonstrated a sigmoid relation between peptide dose and the extent of lysis at one timepoint in an *in vitro* cytotoxicity assay, with a location and steepness that varied with the particular TCR and peptide but over ranges of peptide doses comparable to ours. They showed that as few as two pMHC within the interface between the T cell and its target were sufficient to induce lysis at least in a proportion of contacts, an effect saturating at between 4-200 pMHC, consistent with other studies [39], [40]. Along similar lines, Henrickson *et al.* [33] showed in an LCMV model that a sharp threshold of peptide dose given to dendritic cells (DC) exists for activation of specific CD8 T cells, corresponding to between 30 and 60 pMHC complexes per DC.

If dissociation of peptide from MHC generates the refractory or ‘invisible’ targets in the *in vivo* assay, these results suggest that these targets have reached very low surface densities of specific pMHC. It is then possible that the greater proportion of refractory cells among the T cell targets derives in part from their 2–3 fold lower levels of MHC class I expression (Figure 4). Further, the fact that we and others [24] observe incomplete killing even in the populations receiving high doses of peptide suggests heterogeneity in peptide uptake can be substantial.

### Comparing the efficiency of CTL killing of peptide-pulsed and infected targets

Our work builds on other studies that used the splenic killing assay and exposes different sources of heterogeneity that need to be considered when estimating rates of CTL surveillance. However, the issues that we raise highlight the need for measurements of CTL efficacy performed with live replicating pathogens in relevant tissues, for several reasons. First, there appears to be considerable variation across *in vivo* cytotoxicity assays in the parameters defining CTL activity, likely deriving from differences in microenvironment, TCR specificity, the mode of CD8 T cell priming and hence effector quality, and target cell susceptibility. Second, both the *in vitro* and *in vivo* analyses confirmed earlier findings that peptide dose influences the ability of CTL to detect pulsed targets, but it is not known what peptide doses yield physiologically relevant levels of cognate pMHC on target cells. Third, the influenza infection is well-controlled by the time of the assay 7 days post-challenge; inflammation in the spleen even while killing of peptide-pulsed targets is occurring is presumably low, and so CTL motility and any ability to home to targets may differ between this scenario and one in which an infection is ongoing. Finally, while we have focused on the lytic mode of CTL action, they may also control the spread of intracellular pathogens by non-lytic mechanisms [41], [42] that will presumably not be manifest in assays using peptide-pulsed targets.

### The importance of considering sources of heterogeneity in host-pathogen dynamics

The parameters defining how CTL survey and kill infected cells are key elements of models of the within-host dynamics of intracellular pathogens. Deterministic models assuming homogeneous mixing of components of the immune system and infected cells have been used widely and have provided many mechanistic insights into the progression and control of viral infections (for a review, see for example Ref. [43]). While the functional forms of the terms in these models may be appropriate for describing the dynamics of an infection, the parameters they contain are usually compound quantities and may implicitly average over spatial and cellular heterogeneity. Characterising this heterogeneity is important when attempting to make more detailed quantitative statements regarding host-pathogen interactions. For example, a substantial number of CTL in our *in vivo* assay resided in the red pulp, and would only have been encountered by the proportion of splenocytes that egress from the white pulp over the course of the assay. Depending on the time take to transit the red pulp, it may be that these CTL contribute very little to killing of targets. Estimates of per-CTL killing rates will then be too low if these CTL (enumerated following the homogenisation of whole spleens) are assumed to be co-localised with splenocytes only. With increasing availability of in-vivo imaging data, quantitative immunologists will be able to characterise the within-host ecology of infections in more detail, and specifically the critical sizes of effector cell populations needed for immunity.

## Materials and Methods

### Ethics statement

The UK Home Office Project Licence 80/2506 (Development and function of innate and adaptive immune responses) covers all animal experiments conducted at the NIMR.

### Mice

Ly5.1 C57BL/6J, Ly5.2 C57BL/6J, and F5.Rag1-/- mice were bred and maintained in a conventional pathogen-free colony at the National Institute for Medical Research, London, UK. All lines were of H-2b haplotype. Animal experiments were performed in accordance with UK Home Office regulations.

### Flow cytometry

The following monoclonal antibodies and cell dyes were used: CD45.2 PE-Cy7, CD45.1 FITC, TCR*β* APC, B220 PE-TexasRed (all eBioscience), H-2Db PE (BioLegend), LiveDead nearIR and CellTrace Violet (both Invitrogen), and H-2Db-ASNENMDAM dextramer-PE (Immudex). Samples were acquired on CyAn ADP (Dako Cytomation), Canto-II (BD) or Fortessa X20 (BD) flow cytometers, and analysis was performed with FlowJo software (Treestar).

### Cell culture

Cell culture medium was RPMI supplemented with 10% FCS, 2mM glutamine, 1% penicillin/streptomycin and 50 *µ*M *β*-mercaptoethanol (all Sigma).

### Preparation of target cells

Splenocytes from naive Ly5.1 or Ly5.2 C57BL/6J mice were cultured with NP68 peptide (influenza NP366-374, strain A/NT/60/68, ASNENMDAM, Mimotopes) at 10^−6^ M, 10^−7^ M, 10^−8^ M, 10^−9^ M, or in culture medium alone for unpulsed cells, for 2 hours at 37°C. These cells were then labelled with CellTrace Violet (CTV) at either 10 *µ*M, 2.5 *µ*M, 625 nM, 156 nM or 40 nM, respectively. Following peptide pulse and CTV labelling, target cells were mixed together in equal ratios.

### The *in vivo* cytotoxicity assay

Ly5.1 C57BL/6J mice were injected IV with 2 million lymph node cells from Ly5.2 F5.Rag1-/- mice and A/NT/60-68 influenza virus, to generate a spleen-resident population of NP68 specific CTL. Seven days later, 10 million Ly5.2+ target cells were injected per recipient mouse. At indicated timepoints from 0.5–24 hours after injection of targets, mice were sacrificed and spleen and blood were harvested for analysis by flow cytometry. Care was taken in the timing of both injection and sacrifice for individual mice, and organs were harvested directly into ice cold media, to ensure an error of no more than 5 minutes in the reported timepoints. Target and effector cells were distinguished from host cells by expression of Ly5.2; CTV fluorescence was used to identify target cells that had been pulsed with different doses of peptide, while effector cells were CTV-unlabelled. Staining for TCR*β* and B220 was used to identify T and B cell targets.

### The *in vitro* conjugation and degranulation assay

To generate effector CTL, lymph node cells from Ly5.2 F5.Rag1-/- mice were activated *in vitro* for three days in the presence of NP68 peptide (10^−8^ M). Activated blasts were purified by Ficoll (GE Healthcare) density-gradient centrifugation and expanded for a further four days in the presence of 10 nM IL-2 (Peprotech). Ly5.1+ target cells were prepared as described above. CTL and target cells were added to wells at an E:T ratio of at least 5∶1, and briefly centrifuged to initiate cell contact. Cells were co-cultured at 37°C for the indicated period of time (10 minutes – 2 hours) in the presence of anti-LAMP1a (eBioscience) to detect degranulation of CTL during the culture period. At the end of the culture period, cells were immediately fixed with IC fixation buffer (eBioscience) to preserve E:T conjugates. Samples were then stained and analysed by flow cytometry, with the addition of a known number of AccuCount fluorescent particles (Spherotech) to determine cell counts. Target and effector cells were identified by expression of Ly5.1 or Ly5.2 respectively, and E:T conjugates by dual fluorescence for these markers along with forward scatter area and width characteristics to identify doublets. Staining for TCR*β* and B220 was used to identify T and B cell targets, and CTV fluorescence to identify cells that had been pulsed with different doses of peptide.

### Immunofluorescence

Ly5.1 C57BL/6J mice were injected IV with 2 million lymph node cells from Ly5.2 F5.Rag1-/- mice and A/NT/60-68 influenza virus, to generate a spleen-resident population of NP68 specific CTL. Seven days later, at the time when *in vivo* cytotoxicity assays were performed, mice were sacrificed and spleens harvested for analysis. Each spleen was cut in two, and the weight of each segment recorded. One segment was processed for cell counting and analysis by flow cytometry; the other segment was immediately frozen in liquid nitrogen. Frozen spleen segments were then embedded in OCT compound (VWR International). At least three non-consecutive sections 7 *µ*M thick were cut from each spleen, and stained with antibodies to Ly5.2, IgD and CD4 (all eBioscience). Separate images for each fluorescence channel were collected at 20x magnification on a Leica SP5 confocal microscope, and analysed using ImageJ software (NIH). IgD and CD4 fluorescence was used to manually identify regions of interest corresponding to B cell zones, T cell zones and red pulp, and Ly5.2 fluorescence was subsequently used to enumerate CTL within each of these regions.

### MHC-I turnover assay

Single cell suspensions were prepared from the spleen of C57BL/6J mice and incubated at 37°C in culture medium for the indicated periods of time in the presence of 5 *µ*g/mL Brefeldin A (Sigma) or vehicle control (DMSO, Sigma). Cells were then washed with PBS and stained for TCR*β*, B220 and H-2Db for analysis by flow cytometry.

### Modeling of the *in vivo* cytotoxicity assay

Ordinary differential equation models, described in Results, were used to simulate the flux of peptide-pulsed and unpulsed splenocytes from blood into the spleen and the killing of pulsed targets within the spleen. Parameters were estimated separately for T and B cell target populations at each peptide dose by fitting these models to the logit-transformed fractional killing 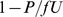, where 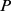 and 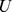 are the numbers of pulsed and unpulsed transferred cell populations recovered from the spleen. The correction factors 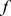 were close to unity and were the ratio of each peptide-pulsed population to the unpulsed population in the inoculum. Estimates of 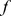 were obtained from the transfer of targets taken from the prepared splenocyte population into naive animals, and observing the proportions of the different target cell populations as they flowed into the spleen. Closed-form solutions to the models were obtained using *Mathematica* [44] and fitted to the data using the nls function in 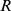 [45]. Data were logit-transformed to ensure the normality and heteroscedasticity of the distribution of residuals. Arcsin square root, complementary log-log and probit transforms yielded similar parameter estimates and qualities of fit.

All data used in this manuscript are provided as Supporting Information (Data S1).

## Supporting Information

Data S1 **Experimental data.** Zipped file containing data from all experiments, in CSV format.
(ZIP)

Text S1 **Supporting Information, sections A–G.**
(PDF)
