# Supplementary material for: Spatial Heterogeneity and Peptide Availability Determine CTL Killing Efficiency In Vivo
Source: PLoS Comput Biol. 2014 Sep 18;10(9):e1003805. doi: 10.1371/journal.pcbi.1003805 (PMC4168979; doi:10.1371/journal.pcbi.1003805)
Supplement: Text S1 — Supporting Information, sections A–G. (PDF) [file pcbi.1003805.s002.pdf]

Supporting Information

A The interpretation of the rate constant k

Under mass-action, we denote the total rate of loss of pulsed targets I due to CTL C as kCI. So kC has dimensions time^{-1}, and $1/(kC)$ is the expected time for a target to meet a CTL. If C and I are measured in the same units, then by symmetry $1/kI$ is the mean time for a CTL to find its next pulsed target. More generally, $1/(k\rho)$ is the mean time to find the next cell in a population of density ρ. The units of ρ determine the units of k, and thus its interpretation. We are free to choose dimensionless units such that the average density of splenocytes is $\rho = 1$, and densities of subpopulations in the spleen are then expressed as proportions of total splenocytes. With this choice of units, $1/k$ has the dimensions of time and is the mean time for a CTL to locate the next cell in the spleen, of any type. The mean time to locate its next target is then $1/kI$, where $0 \leq I \leq 1$. Thus if cell numbers are measured as fractions of splenocytes, the constant k can be interpreted as a CTL surveillance rate.

A further step is required to link the surveillance rate to killing. There is a possibility that a CTL may encounter a pulsed target but fail to detect it as such. If lysis is not 100% efficient, and the rate of loss of pulsed targets is written as kCI, the surveillance rate k is then a compound parameter $k = \kappa p$, where κ is the base rate at which CTL move between surveillance events, and p is the efficiency of lysis. We therefore refer to the parameter k as the effective surveillance rate, and kC as the CTL killing rate.

We also note that the use of the form kCI for the rate of loss of targets holds only if CTL are in excess, such that sequestration of CTL in conjugates with targets does not limit the rate of killing, and/or the time taken to lyse a target is much smaller than the average time between encounters $1/\kappa$ [1]. In the experiments discussed in this study, CTL were always in excess of target numbers and so the modeling of the in-vivo assay does not treat this CTL-target handling time.

B F5 cells dominate the NP68-specific response to influenza virus

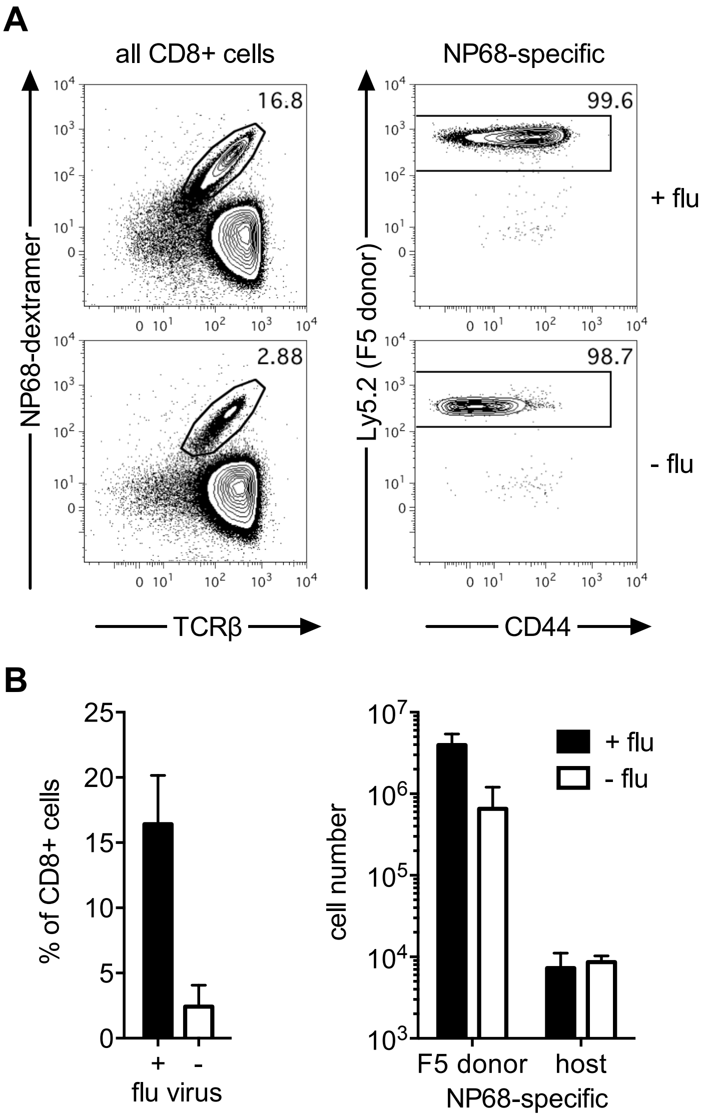

F5 T cells dominate the host response to influenza challenge. Ly5.1 C57BL6/J mice were injected IV with 2 million lymph node cells from Ly5.2 F5.Rag1KO mice, in the presence or absence of A/NT/60-68 influenza virus. Mice were sacrificed seven days later, and cells from the spleen were analysed by flow cytometry. Representative FACS plots (A) were first gated on CD8+ cells, and the proportion of cells expressing a TCR specific for the NP68 dextramer was determined (left panels). Within this NP68-specific population (right panels), donor F5 cells were identified by expression of Ly5.2, and activation status was determined by expression of CD44. Numbers indicate the percentage of cells within each gate. Graphs in (B) show the mean plus standard error of the percentage of all CD8+ cells that were labelled by the NP68 dextramer (left panel; at least four mice per group), and the total number of NP68-specific cells that were either F5 donor or host derived in the spleen of each mouse (right panel).

C Quantifying enrichment for unpulsed cells in blood, and non-specific loss or egress of transferred cells from the spleen

Ratios of pulsed to unpulsed cells in the blood were measured between 30 and 240 minutes post-transfer. The upper panels in the figure below show the fits of a linear model to the logarithm of the pulsed/unpulsed ratio, such that $P/U \sim \exp(-\varepsilon t)$. Enrichment for unpulsed cells in both populations was significant ($p < 0.0004$) and more rapid for T cells (half-life $\log(2)/\varepsilon = 260$ min, 95% CI (200,360)) than for B cells ($\log(2)/\varepsilon = 680$ (430, 1700)). The lower panels show the kinetics of unpulsed cells in the spleen, as they ingress rapidly and are lost more slowly either due to egress or death in the spleen. This kinetic was modeled with Equation (3) in the main text:

$$\frac{\mathrm{d}U_{\mathrm{spleen}}}{\mathrm{d}t} = \sigma N U_{\mathrm{blood}} - \varphi U_{\mathrm{spleen}} = \sigma N U_{\mathrm{blood}}(0) e^{-\lambda t} - \varphi U_{\mathrm{spleen}} \tag{1}$$

where $\lambda = \sigma + \delta$, the total rate of loss of transferred unpulsed cells from the blood. We estimate λ and φ by fitting the above to the timecourse of the frequencies of unpulsed cells in the spleen. The half-lives of loss of unpulsed T and B cells from the blood were not significantly different (T cells, $\log(2)/\lambda$ = 67 (42, 110) mins; B cells, 77 (52, 120) mins). The non-specific loss/egress from the spleen was much slower, with half-life 15 (11, 21) hours for T cells and 25 (17, 41) hours for B cells. This difference is at least qualitatively in line with reports that T cells recirculate through the spleen more rapidly than B cells [2].

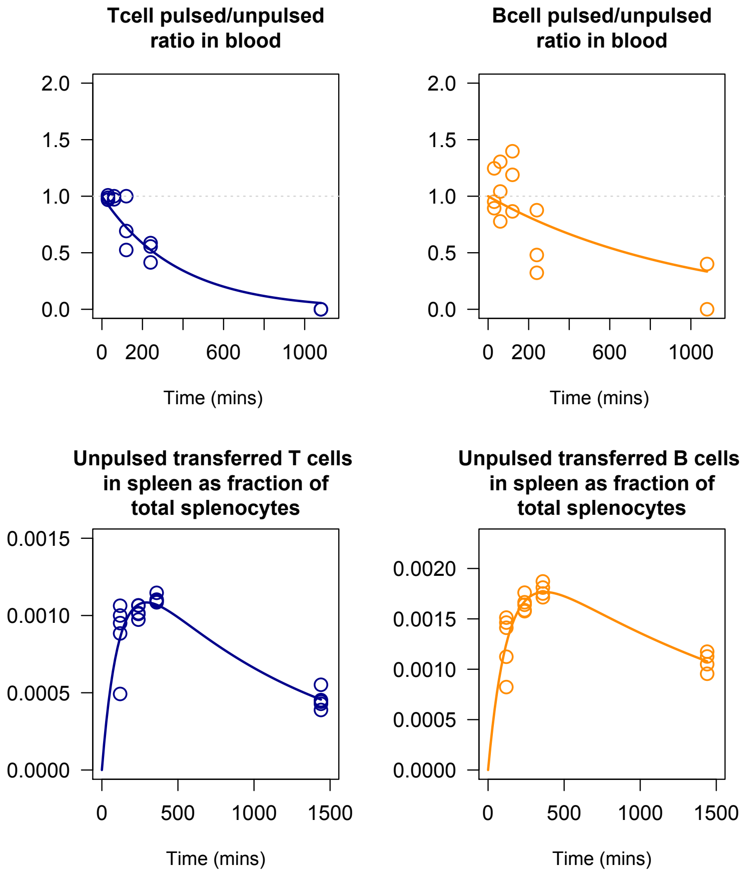

Upper panels: progressive enrichment for unpulsed cells in the blood. **Lower panels**: kinetics of unpulsed cells in the spleen.

D Models

Decay model

Peptide pulsed targets P are assumed to progressively lose susceptibility;

$$\frac{\mathrm{d}U_{\text{spleen}}}{\mathrm{d}t} = \sigma N\, U_{\text{blood}}(0)e^{-(\sigma+\delta)t} - \varphi U_{\text{spleen}}(t) \tag{2}$$

$$\frac{\mathrm{d}P_{\text{spleen}}}{\mathrm{d}t} = \sigma N\, P_{\text{blood}}(0)e^{-(\sigma+\delta+\varepsilon)t} - (K(t)+\varphi)P_{\text{spleen}}(t) \tag{3}$$

where σ is the per capita rate of flow from blood into spleen, δ is the rate of loss of unpulsed cells from the blood into other organs, ε is the excess rate at which pulsed targets are lost from blood into other organs, φ is the rate of non-specific loss or egress of both unpulsed and pulsed cells, $K(t)$ is the (time-dependent) per capita rate of killing of pulsed targets in the spleen, f is the initial ratio of pulsed to unpulsed cells in the inoculum, and N is an unspecified scaling constant that relates the units of measurement of cells in the blood to those in the spleen. Susceptibility falls exponentially once killing commences,

$$K(t) = \begin{cases} 0 & \text{if } t \leq T, \\ K_0 e^{-\mu t} & \text{otherwise.} \end{cases} \tag{4}$$

The fractional killing corrected for the inoculum ratio $f = P_{\text{blood}}(0)/U_{\text{blood}}(0)$ is

$$1 - \frac{P_{\text{spleen}}}{f\, U_{\text{spleen}}}. \tag{5}$$

The populations U_{spleen} and P_{spleen} are each governed by a first order differential equation of the form $dy/dt = \sigma N g(t) - h(t)\, y(t)$ (eqns. 2 and 3), which has solution $y(t)$ proportional to the unknown constant σN. This constant therefore disappears when forming the ratio $P_{\text{spleen}}/U_{\text{spleen}}$. The fractional killing is then a function of three unknowns K_0, T and q and can be identified from the (analytic) solutions to Equations 2 and 3. Solutions were generated in *Mathematica* [3] and parameters were estimated using the `nls` function in *R* [4].

Hidden-target Model

Only a proportion q of peptide-pulsed cells are susceptible. Susceptible cells (S) and cells that are either refractory to killing or who will migrate to areas of the spleen inaccessible to CTL (H) flow in from the blood at rates proportional to qP_{blood} and $(1-q)P_{\text{blood}}$ respectively. Surviving cells remain in their respective states for the duration of the assay.

$$\frac{\mathrm{d}U_{\text{spleen}}}{\mathrm{d}t} = \sigma N\, U_{\text{blood}}(0)e^{-(\sigma+\delta)t} - \varphi U_{\text{spleen}}(t) \tag{6}$$

$$\frac{\mathrm{d}S_{\text{spleen}}}{\mathrm{d}t} = \sigma N\, q\, P_{\text{blood}}(0)e^{-(\sigma+\delta+\varepsilon)t} - (K_0+\varphi)S_{\text{spleen}}(t) \tag{7}$$

$$\frac{\mathrm{d}H_{\text{spleen}}}{\mathrm{d}t} = \sigma N\,(1-q)\, P_{\text{blood}}(0)e^{-(\sigma+\delta)t} - \varphi H_{\text{spleen}}(t) \tag{8}$$

K_0 is zero for $t < T$. The fractional killing is

$$1 - \left(\frac{S_{\text{spleen}} + H_{\text{spleen}}}{f\, U_{\text{spleen}}}\right). \tag{9}$$

Hybrid Model

Here peptide-pulsed cells S transition into a non-susceptible state H at rate ν. Since this transition can occur both in the blood and the spleen, we write the equations for both compartments in full:

$$\frac{\mathrm{d}U_{\mathrm{blood}}}{\mathrm{d}t} = -(\sigma+\delta)U_{\mathrm{blood}}(t) \tag{10}$$

$$\frac{\mathrm{d}S_{\mathrm{blood}}}{\mathrm{d}t} = -(\sigma+\delta+\varepsilon+\nu)S_{\mathrm{blood}}(t) \tag{11}$$

$$\frac{\mathrm{d}H_{\mathrm{blood}}}{\mathrm{d}t} = \nu S_{\mathrm{blood}}(t) - (\sigma+\delta)H_{\mathrm{blood}}(t) \tag{12}$$

$$\frac{\mathrm{d}U_{\mathrm{spleen}}}{\mathrm{d}t} = \sigma N\, U_{\mathrm{blood}}(0)e^{-(\sigma+\delta)t} - \varphi U_{\mathrm{spleen}}(t) \tag{13}$$

$$\frac{\mathrm{d}S_{\mathrm{spleen}}}{\mathrm{d}t} = \sigma N\, S_{\mathrm{blood}}(0)e^{-(\sigma+\delta+\varepsilon+\nu)t} - (K_0+\nu+\varphi)S_{\mathrm{spleen}}(t) \tag{14}$$

$$\frac{\mathrm{d}H_{\mathrm{spleen}}}{\mathrm{d}t} = \sigma N\, S_{\mathrm{blood}}(0)\frac{\nu\left(e^{-(\sigma+\delta)t} - e^{-(\sigma+\delta+\varepsilon+\nu)t}\right)}{\varepsilon+\nu} + \nu S_{\mathrm{spleen}}(t) - \varphi H_{\mathrm{spleen}}(t) \tag{15}$$

where in eqn. 14 we have used the solution of eqns. 10 and 11 for $H_{\mathrm{blood}}(t)$. Again, K_0 is zero for $t < T$, and the fractional killing is

$$1 - \left(\frac{S_{\mathrm{spleen}} + H_{\mathrm{spleen}}}{f\,U_{\mathrm{spleen}}}\right). \tag{16}$$

E Effector:target ratios in vivo

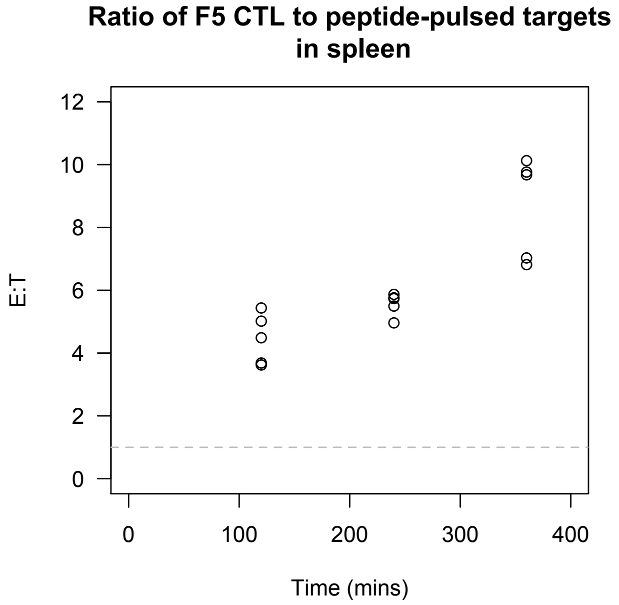

Spleen-averaged effector-to-target (E:T) ratios were greater than 3 for all time points in the *in vivo* cytotoxicity assay. E:T ratios were in the range (29,67) at the last (24h) timepoint when pulsed targets were substantially depleted (not shown).

F No signature of mass-action killing in the spleen

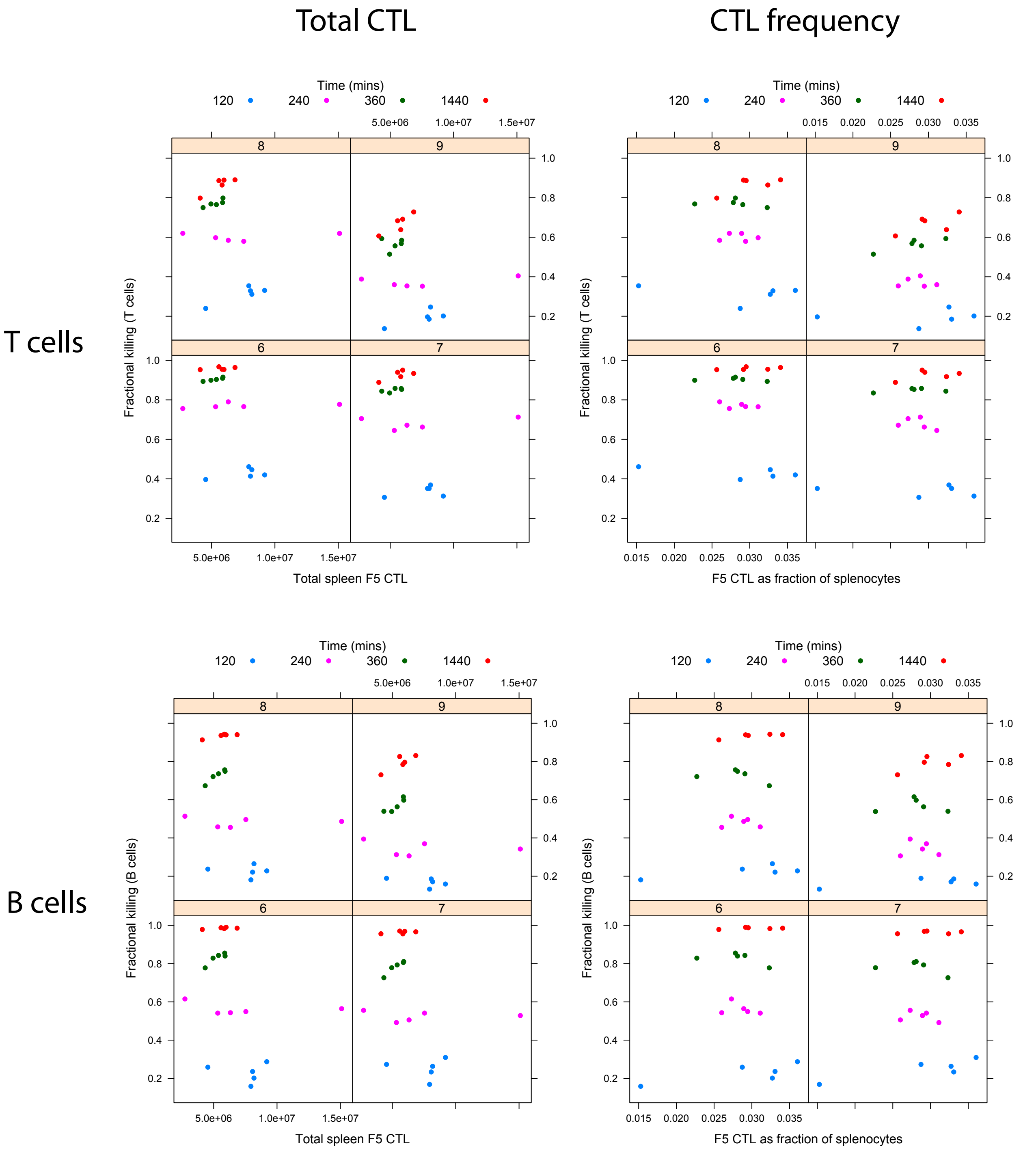

No detectable positive correlation between CTL abundance and the extent of target cell killing. For each target cell type, dose (labels 6-9, indicating -$\log_{10}$(peptide dose)) and timepoint (coloured dots) we found no evidence for mass-action killing kinetics at the whole spleen level after correcting for multiple comparisons (p>0.05), either for CTL measured as total numbers (left column) or as a fraction of total splenocytes (right column).

G Multi-cell conjugates in the *in vitro* cytotoxicity assay

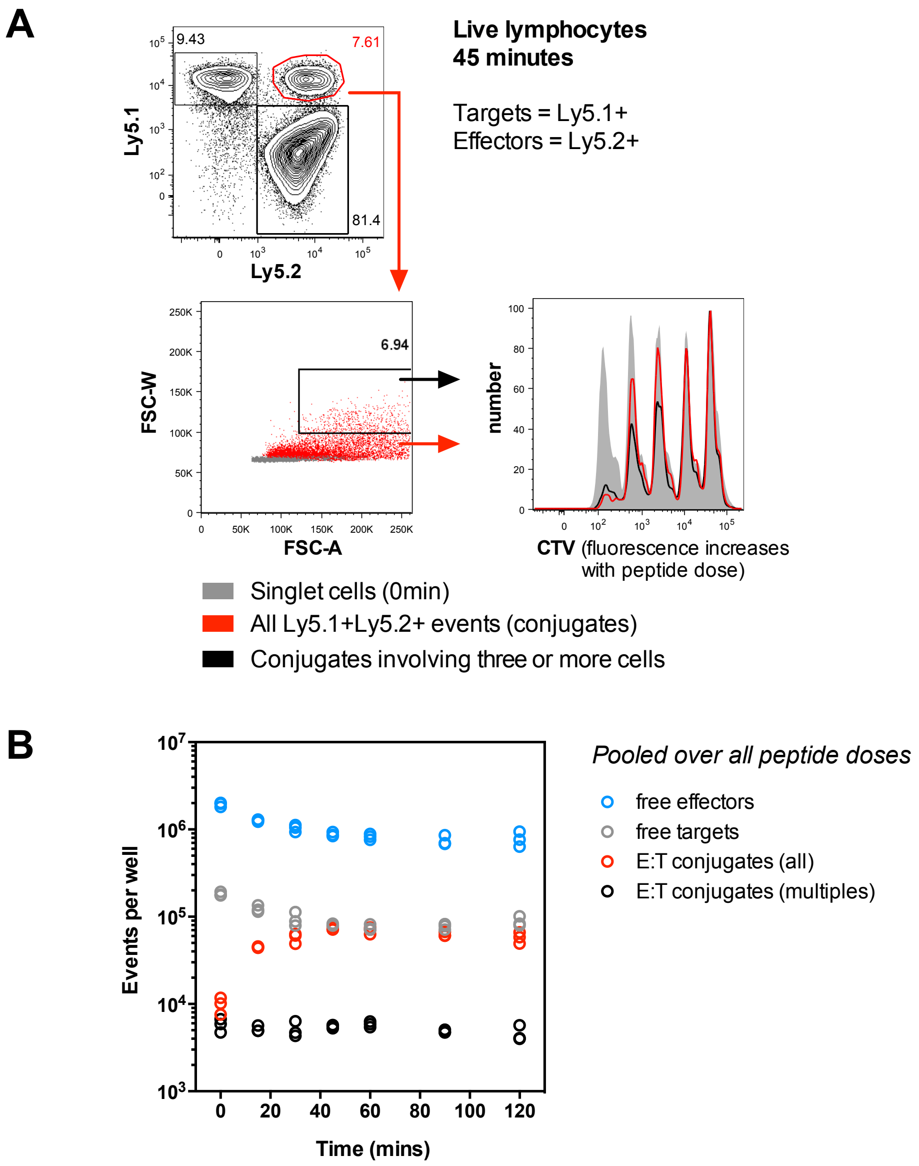

(A) Example flow cytometry data from in vitro culture at the 45 minute timepoint. In all cultures, effector cells were Ly5.2+ and target cells were Ly5.1+. E:T conjugates were identified as events positive for both markers. FSC-A and FSC-W characteristics were used to identify conjugates consisting of 2 cells (1 target : 1 effector). Conjugates involving multiple cells (>2) were also identified. Since effector cells were in excess in these assays (B), it is likely that these multiples consist of two or more effectors bound to a single target. However, the exact number and identity of individual cells within these multiples could not be determined from the available data. Multiples accounted for approximately 10% of all conjugates, and their numbers remained roughly constant over the course of the assay.

References

1. Yates AJ, Van Baalen M, Antia R (2011) Virus replication strategies and the critical CTL numbers required for the control of infection. PLoS Comput Biol 7: e1002274.

2. Steiniger B (2001) Spleen, John Wiley & Sons, Ltd. doi:10.1038/npg.els.0000900.

3. Wolfram Research, Inc (2012) Mathematica 9.0. Champaign, Illinois.

4. R Development Core Team (2013) R: A Language and Environment for Statistical Computing. R Foundation for Statistical Computing, Vienna, Austria. URL http://www.R-project.org.
